# Hospital and Institutional News

**Published:** 1915-03-20

**Authors:** 


### HOSPITAL AND INSTITUTIONAL NEWS.

### THE D.S.O. FOR AN ARMY DOCTOR.

The previous honours lists have shown the gallant as well as valuable part played by the Royal Army Medical Corps in the operations in France, and the latest list, published on March 11, contains among those officers honoured by appointment to be Companions of the Distinguished Service Order "in recognition of their gallantry" the name of Lieut. Eric Christian Lang, R.A.M.C., attached 1st Battalion Leicester Regiment. The following is the official account of his services: "For conspicuous gallantry and devotion to duty on two occasions, especially on February 9, 1915, at Rue Du Bois, in rescuing a severely wounded officer under very difficult circumstances whilst in full view of the enemy." The above rightly speaks for itself, and serves also as a useful reminder of the self-controlled heroism demanded of Army doctors in the field.

### HOSPITAL PROCEDURE AND MILITARY PATIENTS.

Hospital managers will note with interest the points of procedure concerning the reception of soldiers at the London Hospital, which are contained in the article appearing on page 555. Contributed by a correspondent with special access to the facts, the information should prove of value to other hospitals, whose arrangements for undertaking similar work have yet to bear the test of practice. It will also be noticed that the following matters of special interest are recorded. The clothing, uniforms, and underwear required by the patients are supplied by the military authorities, the bales being stored at the hospital and a strict account of all demands being kept. The dental treatment given is specially paid for by the War Office. The cases sent from the hospital to the convalescent homes remain under the control of the hospital's authorities, with the result that procedure is much simplified since permission and correspondence in connection with each case are not required. A similar simplification, also of much value, is the permission accorded to the hospital to issue railway passes to the men on their discharge. This summary should prove not only informing, but suggestive to other institutions likely soon to be busily engaged in similar work and desiring to have the necessary procedure simplified as much as possible.

### TENDERS IN WAR-TIME.

The difficulties which have been experienced, on both sides, over hospital contracts for supplies and stores have led St. Bartholomew's Hospital to take the extreme step of declining tenders for all commodities except milk. The ordinary contract for a six months' supply was recently replaced by an invitation for tenders extending over a period of three months, owing to the general rise in prices. The authorities thereupon came to a decision that no tenders for meat, bread, butter, cheese, and eggs should be accepted. In the case of milk a tender for a twelve months' supply was agreed to. At present, therefore, the hospital will be purchasing the above supplies at practically market prices; but should prices fall, tenders again will probably be called for. As prices are at present the increase in the cost of a year's provisions would amount to some £4,000 or £5,000; and the latest contract price for milk, of which the daily consumption averages 200 gallons, shows an increase in cost amounting to £650 a year. It will be interesting to see from this experience how the new plan succeeds, for the departure from the contract system is one of the remarkable changes occasioned by the war.

### THE REGIMENTAL STRETCHER-BEARERS.

The British success at Neuve Chapelle last week is made specially interesting to hospital workers from the fact that the "descriptive account" afterwards furnished by the "Eye-Witness" present with General Headquarters pays a striking tribute to the fine work and gallantry of the bearer parties under fire. The following extract speaks for itself: "All the wounded have borne testimony to the extraordinary devotion and gallantry of the regimental stretcher-bearers and the bearer parties who have worked till they dropped from sheer exhaustion under the hail of shrapnel and machine-gun fire which swept the open fields during the advance." The message is also significant in the statement which it makes as to the spirits not merely of the troops, but of the wounded, which have been astonishingly raised by the success of this advance. It can be well understood that any change from the monotonous trench warfare must be welcome, and it is good to know that the wounded have brought to hospital a feeling which helps to counterbalance their injuries.

### DISTRICT MEDICAL OFFICERS AND SECURITY OF TENURE.

A dispute has arisen between the Guardians of the Leighton Buzzard Union and the Local Government Board as to the conditions of appointment of the district medical officers in the Union. The Guardians wrote asking for sanction to make the appointments annual ones, and have been asked to give reasons for their wish to depart from the usual procedure. In the discussion which arose it was said that resolutions embodying the Guardians' reasons had already been sent, and that the Local Government Board was evidently resolved to insist upon permanent appointments being made. It was finally decided to defer the matter in order that the Clerk might submit the Poor-Law Orders dealing with it. The Medical Appointment Order, 1857, is quite clear. Article 2 states that every district medical officer duly qualified and resident within his district shall hold office until he die, resign, be proved insane, become legally disqualified to hold office, be removed by the Local Government Board, or cease to reside within his district. Article 6 provides that Guardians may make temporary appointments in cases of emergency or under special circumstances, if the Local Government Board approves.

### THE EVIL OF ANNUAL APPOINTMENTS.

THE Local Government Board has always required substantial reasons before sanctioning any departure from these terms of appointment. The district medical officer in carrying out his duties may easily find himself in conflict with a majority of his Guardians, and it is essential for his freedom of action that he should feel that he cannot be dismissed arbitrarily. The fundamental objection to temporary appointments is that it enables Guardians quietly to get rid of a medical officer at the end of his year of office without giving any reason for their action, and without the medical officer having any appeal against their decision. The possibilities of abuse inherent in the system are obvious, and have been illustrated frequently in the case of medical officers of health of small districts whose appointments are annual ones.

### SWANSEA HOSPITAL AND THE WAR.

THE Rev. H. C. Mander has been elected chairman of the Swansea Hospital for the ensuing year in succession to Dr. Elsworth. The new chairman took the opportunity to remark on the value of voluntary institutions to the country, and declared that the Swansea Hospital would be prepared even to treble the fifty beds which had been placed at the disposal of the military authorities.

### THE TEACHING OF SEX HYGIENE.

THE Report of the London County Council for 1913 contains a note on the decision of the Education Committee in May, 1913, not to make the teaching of sex hygiene a class-subject in public elementary schools. This decision was based on the adverse report of a special sub-committee which received evidence from a number of expert witnesses. The sub-committee point out that reticence and modesty are great safeguards; and that collective teaching is prejudicial by tending to break down these natural barriers. Another evil referred to is the serious mental results which may follow from exaggerated teachings of the physical effects resulting from vicious sexual practices. The Report advocates private talks with individual children by head teachers or school doctors, and the giving of instruction in secondary schools and training colleges. Most medical men will entirely agree with the sub-committee. Whatever one's opinion of Freud's work as a whole, there can be no doubt that he has done good service in drawing attention to the great potentialities for evil contained in the sexual element. The longer that element remains latent in the development of the individual to the adult stage the better. The danger of teaching sex hygiene in childhood lies in the risk of arousing this latent element. The better plan consists in unceasing and unobtrusive vigilance on the part of the child's parents or guardians, and a development of the child's physical activities. No steps should be taken until there are signs of danger. Then is the time for information to be imparted, and the parent must decide who can best give it. In most cases the parents will feel that they cannot, and the task would be better entrusted to the family doctor, or to some close friend or relative.

### THE MIGRATION OF HOSPITALS.

THE punishment meted out by the Navy to the German squadron which bombarded the East Coast on the last occasion on which an attempted raid was reported has apparently checked any repetition. But an interesting incident of these raids was not merely the insurance policies which were taken out by hospitals, but the decision actually to vacate hospital buildings the site of which exposed them to hostile fire. A prominent example of this was the Royal Aberdeen Hospital for Sick Children, the authorities of which decided to remove their patients to a safer place inland, owing to the exposed position of the institution. This decision was enabled to be carried out through the generosity of the trustees of the late Mr. Thomas Ogilvie, who offered Kepplestone House, Rutislaw, rent free. They have now enjoyed nearly three months in their safe quarters, and the Aberdeen Royal Infirmary has undertaken to assist in treating the out-patient children who, of course, could not also be removed. The building fund for the new hospital now amounts to some £12,500, but this sum is far below what is required before a start can be made with the building. It would not have been easy a year ago to conceive a sufficient reason for the migration of this or any other hospital, but now that this has become a *fait accompli*, it throws a curious and sinister light on the recent bombardments.

### NEW PROFESSOR OF PATHOLOGY AT MANCHESTER.

PROFESSOR HENRY ROY DEAN, M.D., F.R.C.P., Professor of Pathology at Sheffield, has been appointed to fill the Chair of Pathology and Pathological Anatomy at Manchester. It will be recalled that Professor Dean has held his late post for two and a half years, and that his research work has received official recognition by a Government grant, which was made to the University of Sheffield to enable him to continue it. Since the declaration of war Professor Dean has served as Major in the Royal Army Medical Corps (T.) at the 3rd Northern General Hospital. After graduating in arts and medicine at Oxford, he took his M.D. degree in 1912, and became a Fellow of the Royal College of Physicians in the following year. His previous appointments have included that of assistant bacteriologist at the Lister Institute.

### THE CANADIAN EMERGENCY HOSPITAL.

ONE of the remarkable emergency hospitals which have lately sprung up all over the country is that established in the grounds and covered tennis court of Cliveden, Taplow, Buckinghamshire, through the action of Mr. and Mrs. Waldorf Astor. On the invitation of Colonel Hodgetts, Chief Commissioner of the Canadian Red Cross Society, the institution was lately inspected by Dr. R. Fox-Symons and Mr. Edmund Owen. The main institution, known as the Canadian Hospital, Cliveden, is divided into four wards, two of which contain twenty-six and two twenty beds. There are also two small wards, one of which is a gallery, containing six and eight beds respectively. Stores, convalescent accommodation, and recreation rooms have been installed, and a hut is being built to house a Thresh steam disinfector. The staff consists of Colonel Charles R. Gorrell, Canadian Medical Service, four assistant medical officers, a matron, and sixteen trained nurses. It is also proposed to build a series of huts capable of accommodating 500 patients. The accommodation also comprises an *x*-ray installation, a pathological laboratory, and a dispensary.

### GERMAN HOSPITAL WARDS.

In view of the natural interest taken in German hospital organisation under the present stress, interest attaches to the following paragraph which appeared in the *Times* from a citizen of a neutral State who had recently been visiting German institutions. There are one or two curious facts also in regard to the way in which the patients' point of view is studied by the German hospital staffs. "On arriving at the hospital of destination the men are washed, shaved, and given fresh clothing. . . . If a patient has received the Iron Cross or has been recommended for such distinction, the fact is stated in large letters on a tablet attached to his bed. 'A man with the Iron Cross never dies if he can help it,' a doctor remarked to me as he pointed to a young boy, a volunteer of eighteen, who was recovering in spite of eight serious wounds. This psychology of the sick-room is, I believe, a matter of special study with the Germans. Their hospital wards are invariably cheerful and pleasant, and the great majority of the *Krankenpflegerinnen* (trained nurses) are trim, comely-looking girls, whose neat costumes give them an air of coquetry deemed very helpful to patients, as an austere-looking surgeon informed me with great seriousness." It will be recalled that in a recent article in The Hospital, containing a description of a German hospital in Hamburg, the writer drew attention to the elaborate and, in his view, glaring and curious colour-scheme adopted in some of the wards with a view to cheering the patients.

### THE CAUSE AND CURE OF PERFUNCTORY FIRE DRILLS.

As a rule, even at the present day, fire drills at hospitals are perfunctory affairs in which few members of the staff take much interest. Sporadic attempts have been made to improve matters, and it is noteworthy that, so far as hospitals are concerned, it was as the result of a paper read by Dr. Clement Dukes, of Rugby, in 1912, that the National Fire Brigades' Union was induced to take the matter up. The Union accordingly adopted a scheme, the main features of which were the two following recommendations: "At large hospitals it would be an advantage if a brigade were organised among the staff and a resident member of the medical staff could be found able and willing to undertake the duties of chief officer. . . ." And ". . . In order to encourage the staff, competitions for smart handling of fire appliances should be held annually, and small prizes awarded. The system would ensure a desire for constant drill in order to maintain efficiency." The Union's scheme provides examiners and awards certificates and badges to those candidates who pass the examination. The first hospital staff to be examined under the scheme was, fitly enough, that of the Hospital of St. Cross, Rugby, and, as a result of the examination held by Colonel Seabroke, chairman of the National Fire Brigades' Union, every member passed the examination. The candidates comprised the matron, Miss H. Osborne, twelve nurses, and six servants. These facts are surely very suggestive, and show what can be done when a member of the medical staff is awake to the importance of proper fire drills. They also prove the necessity for periodical examinations by a central, outside, but recognised body, if the drills are to be serious and the efficiency maintained. If the practice adopted at St. Cross Hospital could become general, there is no doubt that the efficiency of existing fire precautions would be greatly improved, and that such drills as are held would be much more keenly attended. The National Fire Brigades' Union's scheme should be better known than it is at present, and no doubt the hon. general secretary, Mr. Augustus Slater, would be pleased to explain its details to hospital men desirous of participating in its advantages. The Union's address is 22 Northumberland Avenue, W.C.

### SECRETARY FOR A HOSPITAL IN FRANCE.

Soon after the outbreak of war we commented on the fact that the one hospital person whom nobody seemed to want to assist in organising the new emergency hospitals, other than those of military origin, was the hospital secretary. Last week, however, the following advertisement appeared:—"Secretary required, for Medical Hospital in France, with Headquarters in London, Essentials, previous secretarial experience; fluent French, shorthand, and typewriting and accountancy.—State age, salary required, and references by letter to X. Y. Z." Whether this appointment is advertised because the lack of such an official has proved itself unsatisfactory to the institution concerned is an inference only.

### MR. W. E. GLADSTONE'S PHYSICIAN.

There died last week at Chester Dr. W. M. Dobie, a physician who had numbered among his patients Mr. W. E. Gladstone and the late Duke of Westminster. He attended the former during his last illness, and was one of three medical men whose signatures were attached to the bulletin which announced Gladstone's death on May 19, 1898. A graduate of Edinburgh University, who had studied at Dublin, Paris, and Berlin, Dr.

Dobie had many interests outside his profession, though mainly of a scientific nature. He was a Fellow of the Royal Astronomical Society, and with Charles Kingsley helped to found and became President of the Chester Society of Natural Science and Literature. He was eighty-six years old. He was made an honorary freeman of the City of Chester in 1897.

### HOSPITAL SERVANTS WHO LEAVE WITHOUT NOTICE.

The Lancaster Royal Infirmary has been suffering from that trouble with servants which is so much complained of in the smaller households of the middle class. So bad have things grown through servants leaving without notice that the committee decided that it was necessary to make an example, and summoned a wardmaid who had gone away three days after her engagement for 25s., a month's wages, in lieu of notice. The infirmary committee's counsel said that the defendant was formerly a weaver who had been thrown out of work by the war, and that she had been engaged as a wardmaid at £15 a year. She began her duties on January 25, and left on the morning of the 28th without giving notice. By so doing, he said, she had caused great inconvenience; and, as the infirmary had had considerable trouble lately through servants leaving without notice, the matter was reported to the committee, who, though it might seem rather hard to proceed against the defendant, had no alternative but to bring the case into Court so as to convince wardmaids and others that they could not break their contracts. Letters, he added, had been sent by the defendant saying that she was sorry she had inconvenienced the matron, but that it was quite impossible for her to stand the work. She was unable to pay the 25s., as her wages as a weaver were only 7s. weekly. In another letter she again complained of the hardness of the work. After evidence had been given and the infirmary committee's counsel had remarked that a similar case was entered for the next Court, the judge gave judgment for the plaintiffs for the amount claimed and costs. He added that he did not know what the infirmary would think fit to do after having given the warning to other servants. It is certainly rare for hospitals to appear as plaintiffs in the Courts against their employees, but as the Lancaster Royal Infirmary is probably not unique in the difficulty which it has experienced, we quote the case. Housekeepers are aware that a change of employment from factory work to domestic service is hardly ever a success, and the present instance seems to be a typical example of it.

### NEW HOSPITAL IN GLAMORGANSHIRE.

After being projected for about five years, the stone-laying ceremony of a new cottage hospital at Aberavon, Glamorgan, was performed on Thursday last week. The new building, which will be called the Aberavon, Port Talbot, and District Hospital, will be erected at a cost of £3,333, which excludes the cost of furnishing. It will have two wards, the men's having eight and the women's six beds. In addition to the usual administrative rooms, there will be an operating theatre and a mortuary, and as the hospital site, which has been presented by Sir Arthur Vivian, is nearly two acres in extent, there is room for enlargement. All classes have subscribed, and collections have been regularly taken at the industrial works in the locality. In addition the workmen have subscribed £470 towards a maintenance fund. In the past accident cases have had to be taken to Swansea, and as there are large steelworks, tinworks, collieries, and other industrial workshops in the district, the neighbourhood is entitled to have a hospital of its own. The new hospital is expected to be ready for occupation in six months' time.

### YORK COUNTY HOSPITAL AND SOLDIER PATIENTS.

"An unprecedented announcement," to use the words of Mr. W. F. H. Thomson, chairman of the house committee of York County Hospital, was made at the quarterly court last week. It was impossible, he remarked, to present either the accounts or the report, owing to the continued illness of Mr. Neden, the secretary. The accounts and report, therefore, are deferred to the June meeting, by which time no doubt, as Mr. Neden is going away to recover, both will be presented in due course. The hospital has had an unusual interruption through the illness of its responsible officials, since the Dean of York, who resigned the chairmanship of the house committee to become president, and Dr. Hood have also been ill. As regards the criticisms made to the effect that the admission of soldiers had restricted the benefits accorded to the civilian population, Mr. Thomson said that there was no waiting list, so far as male patients were concerned, and that this was the real point at issue. Even if there were waiting male cases, he was sure that it would be the wish of the subscribers and patients themselves to repay the soldiers for their sacrifices. He pointed out also how great had been the strain on the hospital's staff; that the duties of the residents who joined the Colours had been shared by the honorary staff; and that the absence of a secretary, but for the work undertaken by Mr. J. Bye, would have caused very great difficulty. As regards the reception of soldier patients, we do not think that the desire to be patriotic is enough to solve the question or allay any criticism which may exist. The attitude of the hospital should be that, on consideration, it has been found possible to arrange and treat so many soldiers; and that this number has been decided on after the hospital's obligations to the civil population have been given their due weight. For to say merely that there is no waiting list on the male side, though satisfactory in itself, leads at once to the question as to how the needs of the other patients are being provided for. By having an understood and thought-out policy the hospital's double duty can be fully appreciated by the public.

### THE PAY-WARD MOVEMENT IN SCOTLAND.

We are always glad to record the slow but continual development of the pay-ward system, by which men and women of the middle classes, at present medically unprovided for at times of serious illness, are offered an alternative to the immensely expensive and too often radically inefficient nursing home—an institution which has been tolerated in an unreformed condition far too long. The latest recruit, or intending recruit, to the plan of having pay-wards is the Royal Samaritan Hospital for Women, Glasgow, while a vacant building site is suggested as suitable for a pay-block. Dr. W. D. Macfarlane recently remarked that this was a serious problem which must be faced. They were periodically taking in patients, unable to afford nursing home fees, but not technically poor, and a pay-ward would meet their case. The object of the fee, we are glad to see that he explained, would be not to meet the doctors' charges but the hospital's expenses. The matter has been talked of for a considerable time, and it is, therefore, to be hoped that a definite effort will soon be made to use the vacant ground for this useful purpose.

### ACCOUNTS, AND WORKMEN'S REPRESENTATION AT BRISTOL.

The main developments at the Bristol General Hospital during the past year have been only indirectly due to the war, but they have been none the less important. The chief change has been the adoption of the Uniform System of Hospital Accounts, an important step which has been taken in the first year of the appointment of Mr. T. W. Gregg to the secretaryship of the hospital. Of the wisdom of this step there is, of course, no question, and the wonder rather is that it has been delayed so long, although a change in the *personnel* generally provides a useful occasion for a change in procedure. The war has affected the institution perhaps mainly through a drop of £680 in the income from the nursing institution, a decrease attributed to the large call made upon the private staff by the Admiralty and War Office. The scale of salaries of the nursing staff has been increased, and a significant development has been the election of first one and now a second representative of the working classes upon the hospital's committee. In view of the heart-searchings on this subject which have been evident in Bristol institutions in the past it is satisfactory to learn that, in the words of Mr. Herbert Baker, chairman of the committee, at last week's annual meeting, the working-men's committee, also formed, has worked "most cordially" with the committee of management. At the same time the workpeople's contributions appear to have declined from £2,137 to £1,624, and this fact was pointed to by Mr. Found, who suggested that a remedy for the deficiency would be found in the election of more workers' representatives on the committee. In view of a second representative being placed on the committee within a year, Mr. Found's argument can be met by asking why the representation offered has not been met with an increase in the amount subscribed? No doubt the present times of difficulty have something to do with it, but the time was not very opportune for his argument. The facts at all events, on Mr. Baker's showing, are these: the working men contribute about 15 per cent. of the ordinary contributions, and have two representatives on a house committee of twenty-one.

### CARDIFF INSTITUTE FOR THE BLIND.

Efforts to aid the blind of Wales recently received an impetus by the opening of a new wing of the Cardiff Institute for the Blind, the ceremony being performed by Mr. C. Arthur Pearson, who, in the course of an address, announced that the whole of the Welsh Bible is now to be printed in Braille type. The additional accommodation will consist of four workrooms, storage, and an office and board room, and it is anticipated that the present sixty-seven employees will be gradually increased to about one hundred. The contractor is Mr. James Allan, and the hon. architect Mr. David Morgan, of Cardiff. The £2,000 needed to defray the cost of the new buildings has been obtained, but the loss on the trading account for 1914 was £663. The subscriptions amounted only to £470. Objects impossible of achievement at present, owing to insufficient income, are: A fund whereby those workers who have reached the age of sixty may cease their labours and enjoy a well-earned rest until eligible for the old-age pension; a residential school for the blind; a home for blind women, the majority of whom are in lodgings. The Cardiff Institute gives instruction and remunerative employment to the blind and supplements their actual earnings by bonuses. The secretary and manager is Mr. David A. R. Jeffrey, who is keenly interested in developing the work.

### SURGERY LAMPS AND THE LIGHTING REGULATIONS.

Some misunderstanding having arisen with regard to the illumination behind coloured bottles in chemists' windows and the lighting of red lamps outside surgeries, the special organ of the Pharmaceutical Society requested the Commissioner of Police of the Metropolis to state his views on the question. The Commissioner has, therefore, stated that although red is not considered a desirable colour, owing to its having been proved by aerial observations to be specially visible from above, there is no statutory prohibition against the use of red lights or shades in shop windows. Powerful lights behind red shades or coloured bottles would obviously be against the spirit of the Order as to lights, and would be objected to, but there is no general intention to interfere with the limited amount of diffused light which ordinarily passes through the coloured bottles which are so familiar a sign of the chemist's shop. With regard to red lamps outside surgeries, dispensaries, etc., there is, generally speaking, no objection to the use of such lamps, provided that they are of moderate brightness and that the tops are effectively screened. It is hardly conceivable that any doctor would use a brilliant outside light in these times, but it is satisfactory to know the views of the Commissioner of Police on the subject.

### RIVAL CLAIMS TO A CHARITABLE BEQUEST.

The periodic event of two or more institutions disputing in the Courts their right to a legacy bequeathed to a charity bearing the exact title of neither has been again recalled, if not exactly enacted, before Mr. Justice Warrington in the Chancery Division. A summons was taken out by the executors and trustees of the late Mr. W. Raven, of Leicester, to have determined if they had the power to decide whether a legacy of £1,000 bequeathed by the testator to the National Association for the Prevention of Consumption was intended for the benefit of the National Association for the Prevention of Consumption and other Forms of Tuberculosis, or for the benefit of the Leicester and Leicestershire branch of that institution. Counsel submitted that the trustees had power to decide the point, and added that the will contained the following words: "If any doubt shall arise in any case as to the identity of the institution intended to benefit, the question shall be decided by my trustees, whose decision shall be final and binding on all parties." The two institutions declared there was no doubt! As their views were different, counsel for the trustees apprehended that there must be a doubt, and added that the testator was known to be a contributor to the Leicester branch. The judge said that a clause such as that in the will, which left the discretion to the trustees, would, if permissible, prevent a person who was given a legacy suing to recover. After adding that it was contrary to public policy to deprive persons from resorting to public tribunals to decide their legal rights, he declared the provision in the testator's will to be unlawful, and gave judgment for the National Association for the Prevention of Consumption and other Forms of Tuberculosis.

### DUTY-FREE RECTIFIED SPIRIT IN HOSPITALS?

Our readers will have observed with interest that, before the adjournment of Parliament on Tuesday last week, Mr. Bridgeman, M.P., gave notice to ask the Chancellor of the Exchequer: "If he will consider the desirability of providing in the Finance Bill for the supply of duty-free rectified spirit to hospitals for the making of tinctures under similar restrictions to those which regulate the supply of absolute alcohol free of duty for purposes of research in clinical laboratories and colleges." The answer to this question will be eagerly watched by our readers, who will recall the arguments that were marshalled in favour of this concession in The Hospital of November 21, 1914, p. 181.

### BAGTHORPE INFIRMARY FOR MILITARY PATIENTS.

The following details of a scheme approved by the Guardians for providing accommodation for the sick poor, who will be removed when the military authorities take over the hospital block and sanatorium at Bagthorpe Infirmary, are of interest at the present time. Phthisical patients are to be sent to the City Sanatorium; mental patients are to go to the schools at Sneightondale and Scotland Place respectively, males being accommodated at the former and females at the latter. The casual wards are to be closed to vagrants, and men from the test-house are to be transferred to them. The mental wards and test-house are to be used as hospitals for the sick poor. It is interesting to note in this connection that the number of vagrants to whom relief was granted at the Nottingham Workhouse during the first week of March was only 66, a remarkable drop from the figure of 157 recorded twelve months ago. The Board has received letters of thanks both from the Local Government Board and from the Army Council for handing over the infirmary to the military authorities.

### NEW SECRETARY OF THE ROYAL EYE HOSPITAL.

Mr. G. Norman Taylor has been appointed secretary of the Royal Eye Hospital, Southwark, in succession to Mr. Edwin Easton. Mr. Taylor has been assistant secretary to the Radium Institute since it was opened in 1911, previous to which he held a Civil Service appointment. As he is under thirty years of age, Mr. Taylor's new post should give scope to the abilities which have hitherto shown him a man of promise and capacity, likely to rise to any opportunities which may fall in his way. He is to take up his duties in a week or two's time.

### THIS WEEK'S DRUG MARKET.

Business this week has pursued much the same course as last week; that is to say, the upward tendency of prices generally has been maintained, and the demand has been good. A most remarkable advance in the price of cod-liver oil has taken place, present quotations being something like double those of a fortnight ago; the cause of the rise in value has already been stated—namely, the heavy demand for the Norwegian oil from Germany—and although the present rates are altogether disproportionate to the quantity of oil produced so far this season, it is by no means improbable that the highest figure has not yet been reached. Some nine or ten years ago cod-liver oil sold at more than double the present high value, but this was due to the failure of the fishing, and not to any unusual demand. Acetyl-salicylic acid has also advanced much further in price, and the end of the advance is not yet in sight. Other synthetic drugs whose values continue to tend in an upward direction are phenacetin, phenazone, methyl-salicylate, salicylic acid, and sodium salicylate; while among the drugs which are dearer are cascara sagrada, bromides, and senega. The condition of the market places buyers in an awkward condition, but apparently the only thing to do is to buy sufficient to satisfy reasonable requirements, for at present there seems to be no prospect of a decline in the value of the drugs affected by the war. The opening up of the Dardanelles would, no doubt, effect a reduction in the prices of some few drugs. The advanced price of cocaine is maintained.

